# Case mix, outcomes and comparison of risk prediction models for admissions to adult, general and specialist critical care units for head injury: a secondary analysis of the ICNARC Case Mix Programme Database

**DOI:** 10.1186/cc5066

**Published:** 2006-10-12

**Authors:** Jonathan A Hyam, Catherine A Welch, David A Harrison, David K Menon

**Affiliations:** 1 Department of Neurosurgery, Charing Cross Hospital, London, UK; 2 Intensive Care National Audit and Research Centre (ICNARC), Tavistock House, Tavistock Square, London WC1H 9HR, UK; 3 University of Cambridge, Addenbrooke's Hospital, Cambridge CB2 2QQ, UK

## Abstract

**Introduction:**

This report describes the case mix and outcome (mortality, intensive care unit (ICU) and hospital length of stay) for admissions to ICU for head injury and evaluates the predictive ability of five risk adjustment models.

**Methods:**

A secondary analysis was conducted of data from the Intensive Care National Audit and Research Centre (ICNARC) Case Mix Programme, a high quality clinical database, of 374,594 admissions to 171 adult critical care units across England, Wales and Northern Ireland from 1995 to 2005. The discrimination and calibration of five risk prediction models, SAPS II, MPM II, APACHE II and III and the ICNARC model plus raw Glasgow Coma Score (GCS) were compared.

**Results:**

There were 11,021 admissions following traumatic brain injury identified (3% of all database admissions). Mortality in ICU was 23.5% and in-hospital was 33.5%. Median ICU and hospital lengths of stay were 3.2 and 24 days, respectively, for survivors and 1.6 and 3 days, respectively, for non-survivors. The ICNARC model, SAPS II and MPM II discriminated best between survivors and non-survivors and were better calibrated than raw GCS, APACHE II and III in 5,393 patients eligible for all models.

**Conclusion:**

Traumatic brain injury requiring intensive care has a high mortality rate. Non-survivors have a short length of ICU and hospital stay. APACHE II and III have poorer calibration and discrimination than SAPS II, MPM II and the ICNARC model in traumatic brain injury; however, no model had perfect calibration.

## Introduction

Traumatic brain injury is a common and potentially fatal condition. In the United States, 50,000 people die annually after head injury and 80,000 to 90,000 suffer long-term disability [1]. Head injury accounted for more than 120,000 admissions in England during 2000 to 2001, utilising over 320,000 bed days [2]. Ninety percent of head injuries seen in UK Accident and Emergency departments are mild, defined by the Royal Society of Rehabilitation Physicians as Glasgow Coma Score (GCS) 13 to 15 [3], 5% are moderate (GCS 9 to 12) and 5% are severe (GCS 3 to 8) [4].

Patients with severe head injury, in whom treatment is not deemed futile, are cared for in general or specialist intensive care units (ICUs). This is for a variety of reasons, most importantly because patients with a GCS below 9 need endotracheal intubation to protect their airway patency. Other reasons include management of associated extracranial injuries. Therefore, head injury presents a large burden on critical care facilities in the UK.

Factors associated with increased mortality after head injury include age [5], presenting GCS [6], lower blood pressure [7], serum glucose [8], and hypoxia [9]. Various risk prediction models, such as Simplified Acute Physiology Score (SAPS) II, Mortality Probability Models (MPM) II and Acute Physiology And Chronic Health Evaluation (APACHE) II and III have also been demonstrated to predict head injury mortality [10,11].

This report describes head injury patients admitted to ICUs across England, Wales and Northern Ireland, identified using the Intensive Care National Audit and Research Centre (ICNARC) Case Mix Programme (CMP) Database. The case mix of ICU admissions, outcome and activity associated with these admissions are described. The aim is to indicate the burden of head injury on intensive care nationally to help inform future planning policy and to allow local units to compare their practice and results. A comparison is also made of the ability to predict head injury mortality using several commonly used risk prediction models, which are already well established in intensive care audit.

## Materials and methods

### Case Mix Programme Database

The CMP is a national comparative audit of adult, general critical care units (including ICUs and combined intensive care and high dependency units) in England, Wales and Northern Ireland. Additionally, a small number of specialist units, including neurosurgical units, participate in the audit. The data undergo extensive validation before being incorporated into the CMP Database. Details of the data collection and validation have been reported previously [12]. Data were extracted for 374,594 admissions to 169 general ICUs and 5,743 admissions to two neurosurgical units, from the period December 1995 to May 2005.

### Selection of cases

Primary and secondary reasons for admission to ICU are coded in the CMP Database using the ICNARC Coding Method [13], a hierarchical method specifically designed for coding the reasons for admission to ICU. Admissions were selected from the database if they were aged 16 years or over, and the primary reason for admission to ICU was recorded as 'Primary brain injury', 'Subdural haematoma', or 'Extradural haematoma'.

### Data

Data were extracted on the case mix, outcome and activity as defined below.

#### Case mix

The lowest total GCS from the first 24 hours following ICU admission (or the entire stay, if less than 24 hours) is recorded in the CMP if the admission was not sedated or paralysed and sedated for the whole of the first 24 hours.

The pre-sedation GCS quantifies the level of consciousness following traumatic brain injury before the admission is sedated or paralysed and sedated. It is recorded if the admission was sedated or paralysed and sedated at any time during first 24 hours in ICU.

Severity of illness was summarised by the APACHE II score [14], encompassing weightings for acute physiology (defined by derangement from the normal range for 12 physiological variables in the first 24 hours following admission to ICU), age, and a past medical history of specified severe conditions.

Admissions following emergency surgery were identified based on the source of admission to the CMP unit and the National Confidential Enquiry into Perioperative Death (NCEPOD) classification of surgery, as has been described previously [12].

#### Outcome

Survival data were extracted at discharge from the CMP unit and at ultimate discharge from hospital.

#### Activity

Length of stay in the CMP unit was calculated in fraction of days from the dates and times of admission and discharge. Length of stay in hospital was calculated in days from the dates of original admission and ultimate discharge. Readmissions to the unit within the same hospital stay were identified from the postcode, date of birth and sex, and confirmed by the participating units.

### Analyses

A statistical analysis plan was agreed *a priori*. The analyses performed were as follows.

#### Descriptive statistics

The case mix, outcome and activity, as above, were described for head injury admissions. Continuous variables were summarised by mean and standard deviation, or median and interquartile range for skewed variables.

The number of admissions and mortality were presented by lowest total GCS for admissions not sedated/paralysed and sedated for the entire first 24 hours in ICU, and by pre-sedation GCS for admissions sedated/paralysed and sedated for the entire first 24 hours in ICU.

#### Evaluation of models in head injuries admissions

The prognostic ability of the APACHE II [14], APACHE III [15], SAPS II [16], MPM II [17] and ICNARC [18] models were assessed. Coefficients for APACHE II were taken from the UK-specific model [19]. These models were evaluated for discrimination (the ability of the model to distinguish survivors from non-survivors), calibration (the accuracy of the estimated probability of survival), and overall fit.

Discrimination was assessed by the area under the receiver operating characteristic (ROC) curve (AUC) [20]. Calibration was assessed by the Hosmer-Lemeshow C*-statistic [21] and Cox's calibration regression [22]. Overall fit of the model was assessed by Brier's score [23].

The AUC (also called the concordance statistic) measures the probability that a randomly selected non-survivor has a higher prediction than a randomly selected survivor. A value of 0.5 indicates no discrimination, and 1 indicates perfect discrimination.

The Hosmer-Lemeshow test divides the data into a number of equal-sized groups (typically 10) based on the predicted mortality – that is, the 10% with the lowest predicted mortality, the 10% with the next highest, and so on. The observed mortality in these groups is then compared to the expected mortality predicted by the model. The C*-statistic is calculated by summing the following quantity over these groups:

N × (O - E)^2^/(E × (1 - E))

where N is the number of patients in the group, O is the observed mortality and E is the expected mortality. Under the null hypothesis of perfect calibration (observed mortality = expected mortality), the C*-statistic has a chi-squared distribution with degrees of freedom equal to the number of groups. As the C*-statistic is sensitive to variation in sample sizes, it is not appropriate to directly compare C*-statistics for different models unless they have been calculated on the same patients.

Cox's calibration regression tests for a systematic lack of calibration by performing a linear recalibration of the log odds. The log odds are given by log(*p*/(1 - *p*)), where *p* is the mortality probability. The following model is fitted:

true log odds = slope × predicted log odds + intercept

If the model is perfectly calibrated then the slope will be 1 and the intercept 0, that is, true log odds = predicted log odds. This is tested with a likelihood ratio chi-squared test.

Brier's score, developed in relation to meteorological forecasting, is an overall measure of accuracy. It is the mean square error between outcome and prediction. For perfect predictions, Brier's score will be 0; for constant predictions of 0.5 for every individual, Brier's score will be 0.25. The lower the value of Brier's score, the more accurate the predictions.

Admissions were excluded if they were readmissions of the same patient within the same hospital stay, as outcomes would not be independent, or if they were missing the outcome variable of hospital mortality. In addition, the standard exclusion criteria were applied for each model.

Admissions were excluded from APACHE II if they stayed less than eight hours in the critical care unit, were transfers from another critical care unit, or were admitted for burns or following coronary artery bypass graft. Admissions were excluded from APACHE III if they stayed less than four hours in the critical care unit, or were admitted for burns or following coronary artery bypass graft. Admissions were excluded from SAPS II if they were under the age of 18, admitted for burns or following cardiac surgery, transferred to an ICU in another hospital, missing surgical status, or missing ventilation and oxygenation data. The MPM II model gives a mortality prediction on admission to ICU, and again at 24 hours into their stay. The prediction on admission was used for admissions staying less than 24 hours, with the prediction at 24 hours used for all other admissions. Admissions were excluded from MPM II if they were under the age of 18, admitted for burns or following cardiac surgery, or transferred to an ICU in another hospital. The ICNARC model has no exclusion criteria. Participating units in the CMP may elect not to collect additional data for APACHE III, SAPS II and/or MPM II, so all admissions to these units were excluded from the relevant model(s).

Initially, the evaluation of these models was carried out on all the admissions that met the inclusion criteria for each model. A second evaluation was also carried out on just the admissions that met the inclusion criteria for all the models, allowing more direct comparison of the results. As the ICNARC model was developed using the CMP Database, a further sensitivity analysis was performed, excluding all admissions that had been included in the development dataset for the ICNARC model.

The discrimination of the models was also compared to the discrimination of GCS alone – both the lowest GCS from the first 24 hours and the pre-sedation value – in all admissions with a GCS recorded and the subset of these eligible for all the models. As no prediction of mortality is produced directly from GCS, it is not possible to include GCS in comparisons of calibration.

All analyses were performed using Stata 8.2 (Stata Corporation, College Station, TX, USA).

## Results

### Descriptive statistics

Overall, 11,021 admissions following traumatic brain injury were identified in the database, representing 3.0% of all admissions to these units. The case mix, outcome and activity of these admissions are presented in Table 1. Hospital mortality was considerably higher than ICU mortality (33.5% versus 23%). Of the 938 admissions that died in hospital post first ICU discharge, 74 (7.9%) had all active treatment withdrawn prior to ICU discharge and 176 (18.8%) were discharged for palliative care.

**Table 1:** Case mix, outcome and activity of admissions following traumatic brain injury by type of unit

Measures of case mix, outcome and activity	n (percent), mean (SD) or median (IQR)
Number of admissions, n (percent of database)	11,021 (3.0)
Age (years), mean (SD)	44.0 (19.9)
Reason for admission, n (percent)	
Primary brain injury	6,400 (58.1)
Subdural haematoma	3,631 (32.9)
Extradural haematoma	990 (9.0)
Admission following emergency surgery, n (percent)	2,233 (20.3)
First 24 h GCS^a^, mean (SD)	7.3 (4.5)
Pre-sedation GCS^b^, mean (SD)	6.8 (3.6)
APACHE II score^c^, mean (SD)	14.3 (7.4)
Mortality, deaths (percent)	
ICU	2,534 (23.0)
Hospital^d^	3,453 (33.5)
Length of stay (days), median (IQR)	
ICU	
Survivors	3.2 (1.1–8.1)
Non-survivors	1.6 (0.7–4.0)
Hospital^d^	
Survivors	24 (10–51)
Non-survivors	3 (1–9)

^a^Admissions not sedated for the entire first 24 h. ^b^Admissions sedated for the entire first 24 h. ^c^Excluding admissions staying <eight hours in the ICU. ^d^Excluding readmissions within the same hospital stay. APACHE, Acute Physiology And Chronic Health Evaluation; GCS, Glasgow Coma Score; ICU, intensive care unit; IQR, interquartile range; SD, standard deviation.

**Figure 1:**
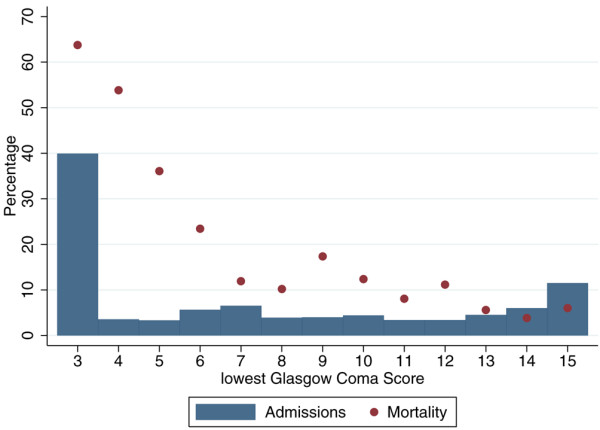
Distribution of Glasgow Coma Score and mortality for admissions with a Glasgow Coma Score recorded in the first 24 hours.

**Figure 2:**
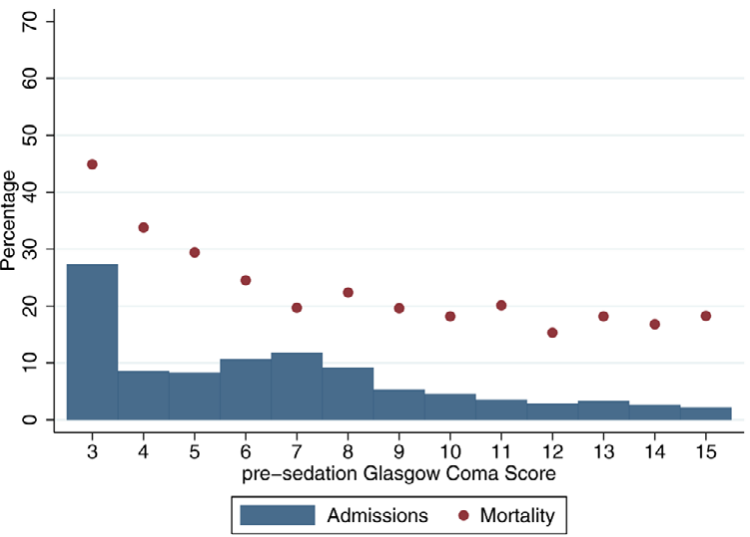
Distribution of Glasgow Coma Score and mortality for admissions with a Glasgow Coma Score recorded before sedation.

Of the 11,021 admissions: 4,766 (43.2%) were not sedated or paralysed for the entire first 24 hours in ICU, and had a GCS recorded during this time; 4,331 (39.4%) were sedated and paralysed for the entire first 24 hours in ICU and had a pre-sedation GCS recorded; the remaining 1,914 (17.4%) did not have a GCS recorded. Figures 1 and 2 show the distribution of GCS and its relationship with mortality for these groups. We see that the relationship between GCS and mortality is more extreme (with a higher mortality for GCS 3 and a lower mortality for GCS 15) for GCS measurements from the first 24 hours in ICU than for pre-sedation measurements.

### Evaluation of models in head injuries admissions

**Figure 3:**
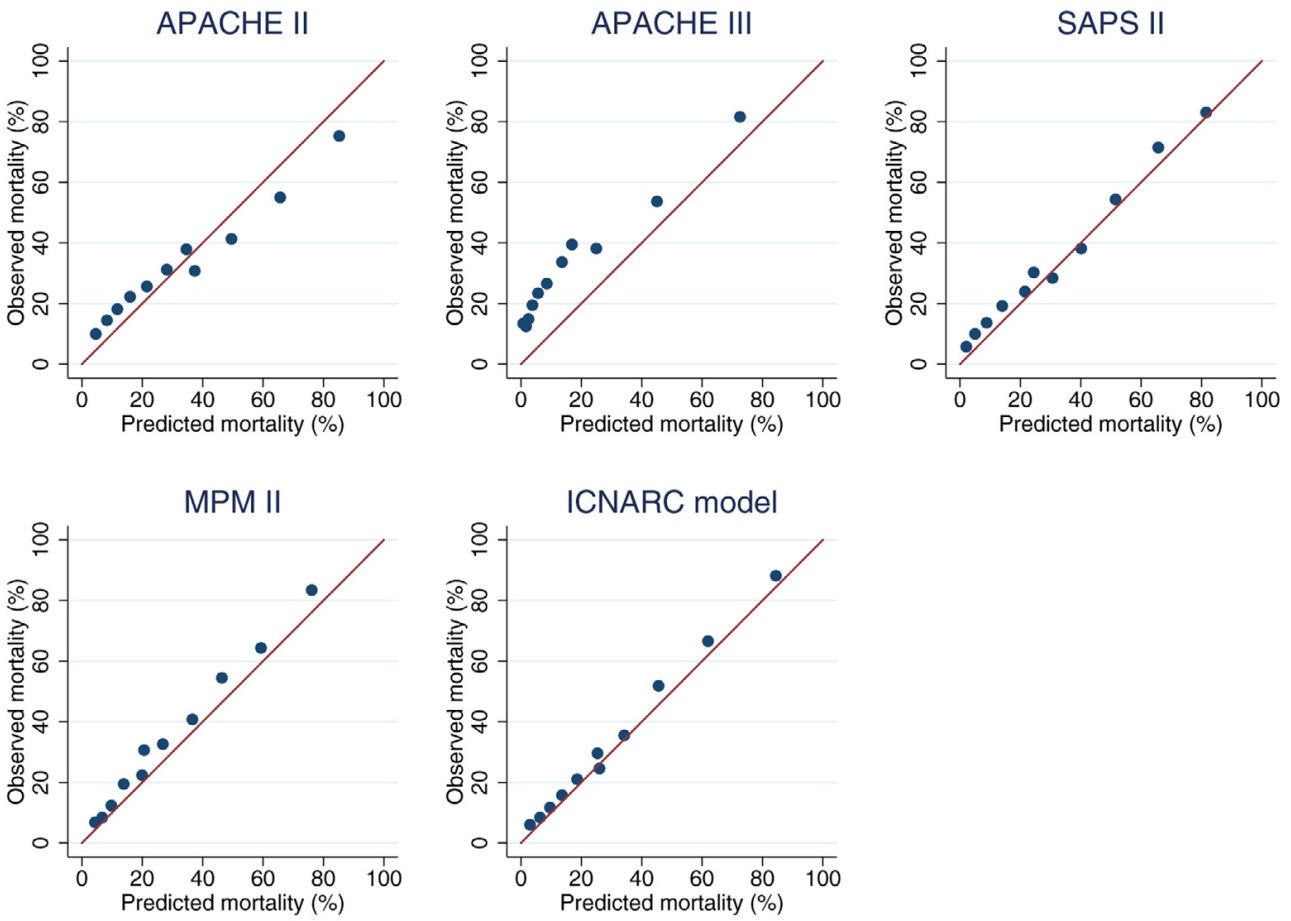
Calibration plots for APACHE II, APACHE III, SAPS II, MPM II and ICNARC models.

**Figure 4:**
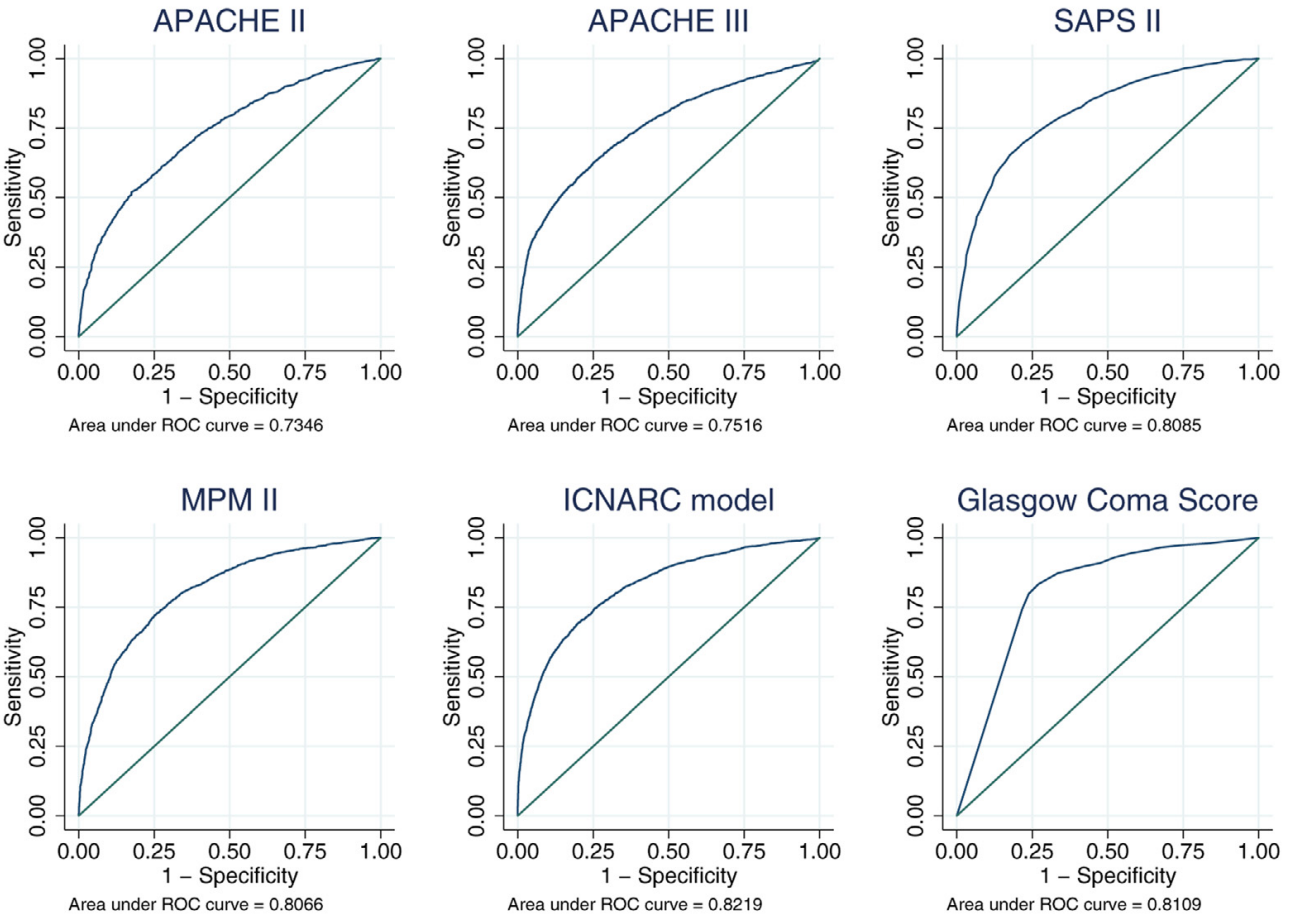
Receiver operating characteristic (ROC) curves for APACHE II, APACHE III, SAPS II, MPM II, ICNARC model and lowest total Glasgow Coma Score.

Tables 2 and 3 show the measures of model performance for each of the five risk prediction models in all admissions eligible for that model and the 5,393 admissions eligible for all five models, respectively. Figure 3 shows calibration plots for the five models, and Figure 4 shows ROC curves for the five models plus lowest total GCS. MPM II excluded the most admissions, with 7,267 admissions included in the analysis, compared to 10,285 for the ICNARC model. However, this was largely due to units electing not to collect data for MPM II. The ICNARC model had the best performance on all measures, closely followed by SAPS II and MPM II. The APACHE models had much poorer performance. The calibration plots (Figure 3) indicate that APACHE III significantly underestimated mortality for all admissions, whereas APACHE II appears to overestimate mortality for higher risk admissions.

**Table 2:** Measures of model calibration and discrimination in all admissions eligible for each model

Model	APACHE II	APACHE III	SAPS II	MPM II	ICNARC
Eligible admissions, n (percent)	8,344 (75.7)	9,021 (81.9)	8,220 (74.6)	7,267 (65.9)	10,285 (93.3)
AUC (95 percent CI)	0.73 (0.72, 0.75)	0.75 (0.74, 0.77)	0.81 (0.80, 0.82)	0.81 (0.79, 0.82)	0.82 (0.81, 0.83)
Hosmer-Lemeshow C*					
χ^2^(10)^a^	316	4542	162	99.5	94.6
p value	<0.001	<0.001	<0.001	<0.001	<0.001
Cox's calibration regression					
Intercept (95 percent CI)	-0.23 (-0.29, -0.18)	0.44 (0.36, 0.52)	0.09 (0.03, 0.15)	0.27 (0.20, 0.34)	0.18 (0.12, 0.24)
Slope (95 percent CI)	0.66 (0.62, 0.70)	0.58 (0.55, 0.62)	0.86 (0.82, 0.90)	1.00 (0.94, 1.05)	0.97 (0.93, 1.01)
χ^2^(2)	262	2,045	74.8	84.8	66.3
p value	<0.001	<0.001	<0.001	<0.001	<0.001
Brier's score	0.187	0.193	0.164	0.168	0.155

^a^Note that due to different sample sizes, the Hosmer-Lemeshow C*-statistic should not be directly compared between models. APACHE, Acute Physiology And Chronic Health Evaluation; AUC, area under the receiver operating characteristic curve; CI, confidence interval; ICNARC, Intensive Care National Audit & Research Centre; MPM, Mortality Probability Models; SAPS, Simplified Acute Physiology Score.

**Table 3:** Measures of model calibration and discrimination in admissions eligible for all models

Model	APACHE II	APACHE III	SAPS II	MPM II	ICNARC
AUC (95 percent CI)	0.74 (0.72, 0.75)	0.76 (0.75, 0.78)	0.81 (0.80, 0.83)	0.81 (0.80, 0.82)	0.84 (0.83, 0.85)
Hosmer-Lemeshow C*					
χ^2^(10)	215	2,478	61.4	38.2	24.9
*p* value	<0.001	<0.001	<0.001	<0.001	0.006
Cox's calibration regression					
Intercept (95 percent CI)	-0.26 (-0.33, -0.19)	0.50 (0.40, 0.60)	0.02 (-0.05, 0.10)	0.20 (0.12, 0.28)	0.17 (0.09, 0.26)
Slope (95 percent CI)	0.66 (0.62, 0.71)	0.61 (0.57, 0.65)	0.89 (0.84, 0.95)	1.04 (0.97, 1.10)	1.08 (1.02, 1.14)
χ^2^(2)	176	1,256	20.3	28.0	17.8
*p* value	<0.001	<0.001	<0.001	<0.001	<0.001
Brier's score	0.187	0.194	0.158	0.163	0.147

N = 5,393 (48.9 percent). APACHE, Acute Physiology And Chronic Health Evaluation; AUC, area under the receiver operating characteristic curve; CI, confidence interval; ICNARC, Intensive Care National Audit and Research Centre; MPM, Mortality Probability Models; SAPS, Simplified Acute Physiology Score.

Excluding all admissions included in the development dataset for the ICNARC model left 2,563 admissions. All models exhibited slightly poorer performance in terms of both discrimination and overall fit in this dataset; however, the order of the models was preserved, with the ICNARC model still demonstrating the best performance.

The discrimination of GCS is compared with that of the risk prediction models in Table 4. While the discrimination of the lowest GCS from the first 24 hours in ICU was good (AUC 0.81) in admissions that were not sedated or paralysed for the entire first 24 hours, it was outperformed by all the risk prediction models. APACHE III, SAPS II, MPM II and the ICNARC model all displayed excellent discrimination (AUC 0.89 to 0.91) in this group. Discrimination of all models was considerably worse when restricted to patients that were sedated and had a pre-sedation GCS recorded; however, the raw GCS still displayed worse discrimination than the models.

**Table 4:** Discrimination of Glasgow Coma Score compared with risk prediction models

	All admissions with GCS recorded		Admissions eligible for all models	
	N	AUC (95 percent CI)	N	AUC (95 percent CI)

First 24 h GCS	4,527	0.81 (0.80–0.82)	2,471	0.81 (0.79–0.83)
APACHE II	3,509	0.85 (0.84–0.87)	2,471	0.85 (0.84–0.87)
APACHE III	3,777	0.89 (0.88–0.90)	2,471	0.90 (0.89–0.91)
SAPS II	3,672	0.89 (0.88–0.90)	2,471	0.89 (0.88–0.90)
MPM II	3,213	0.90 (0.89–0.91)	2,471	0.91 (0.90–0.92)
ICNARC	4,513	0.90 (0.89–0.91)	2,471	0.90 (0.89–0.91)
Pre-sedation GCS	6,684	0.70 (0.69–0.71)	3,745	0.71 (0.69–0.72)
APACHE II	5,671	0.72 (0.70–0.73)	3,745	0.72 (0.70–0.74)
APACHE III	5,573	0.74 (0.72–0.75)	3,745	0.74 (0.72–0.76)
SAPS II	5,159	0.80 (0.79–0.82)	3,745	0.81 (0.80–0.83)
MPM II	4,440	0.82 (0.81–0.83)	3,745	0.82 (0.81–0.83)
ICNARC	6,671	0.80 (0.79–0.82)	3,745	0.81 (0.80–0.83)

APACHE, Acute Physiology And Chronic Health Evaluation; AUC, area under the receiver operating characteristic curve; CI, confidence interval; GCS, Glasgow Coma Score; ICNARC, Intensive Care National Audit and Research Centre; MPM, Mortality Probability Models; SAPS, Simplified Acute Physiology Score.

## Discussion

This study examines the outcomes of 11,021 head injury patients admitted to UK ICUs since 1995, and the predictive ability of five risk-adjustment scores for intensive care head injury mortality.

ICNARC is an independent charity and coordinates a national comparative audit of patient outcomes from participating ICUs. In total, 171 UK ICUs contributed data used in this study: 153 in England, 8 in Northern Ireland and 10 in Wales. The CMP is a high quality database and performs well against the Directory of Clinical Databases criteria [12], comprising data on consecutive admissions from each centre, explicit variable definitions, data collection training for observers and objective variables without scope for inter-observer error.

A limitation of the analysis is that a proportion of admissions, 1,692 patients, did not have a documented GCS/pre-sedation GCS. These patients were, therefore, excluded from the analysis, which may have introduced an element of bias.

Seventy-seven percent of the head injury admissions in this analysis were male, a male to female ratio of 3.3:1. In series of several thousands of head injuries in adults, including patients who did not require intensive care and presented with any GCS, males accounted for 67% to 90% of cases [2,8,24-27]. This association is well established and is correlated with the greater sensation seeking behaviour of males [28]. The mean age of adults admitted to the ICUs in this analysis was 44 years. This is moderately higher than other studies that quote mean ages between 28 and 38 years [8,24-27,29,30].

Survivors' length of stay (LOS) in ICU was a median of 3.2 days. Non-survivors only stayed a median of 1.6 days. The design of this analysis is such that we can only speculate upon explanations for this disparity. Firstly, this may be a reflection of limited provision of ICU beds within participating centres. Much has been written about critical care provision in the UK [31-34]. A shortage of units providing intermediate/high dependency care [35] and intensive care [33] has been identified and appropriately referred patients may still be refused admission to intensive care because of this [34]. Patients in our study may have been cared for on a ward or in a lower level critical care setting and transferred to the ICU only when they had acutely deteriorated, by which point it may have been too late to sizeably influence their outcome. Similarly, there may have been pressure to discharge patients from the ICU prematurely to make way for others with greater perceived critical care need. This appears to be supported by the higher non-survivors' median hospital LOS of three days; that is, patients with ultimately fatal head injury did not spend all of their admission receiving intensive care. Alternatively, the fact that these patients did not stay on an ICU for their entire admission may have reflected sound medical judgement by which intensive care was channelled away from patients whose outcome would not have been expected to be changed significantly regardless of the level of medical attention they received.

A study of 843 head injury patients in a UK ICU demonstrated similar overall median ICU LOS of three days [36]. Asthma patients, in contrast, only stay a median of 1.5 days in UK ICUs and represented only 1.7% of UK ICU admissions [37]. As well as longer ICU LOS, head injury accounted for 3% of all ICU admissions in our analysis. Therefore, with intensive care costing £1,219 to £1,638 per day [33], head injury represents a large burden on critical care resources. The survivors tended to stay in hospital for some time, with a median hospital LOS of 24 days. Overall, median LOS was 23 days in a series of 182 head injury patients presenting with a GCS of less than 9 [38], and 10 days in a series of 843 patients requiring ICU treatment [36]. Asthma and chronic obstructive pulmonary disease median hospital LOS were 8 days [37] and 16 days [39], respectively.

It is notable that non-survivors spent only a median of three days in hospital. It appears that if their injury was serious enough to be fatal, they would die early during the admission, within only a couple of days. In contrast, the long hospital LOS of survivors in our study is not surprising. These are highly dependent patients who need almost all activities of daily living performed for them; those requiring initial intubation will require weaning from the respiratory support and their immobility puts them at risk of multiple medical complications, such as venous thromboembolism and pneumonia. Many of these patients will also have associated injuries that will delay their recovery, such as those to the chest, abdominal organs and spine.

Head injury patients in this analysis had a 77% chance of surviving to leave the ICU and a 66.5% chance of surviving to leave hospital. Our in-hospital mortality rate of 33.5% is comparable to previous studies, whose patients were adults and all received intensive care, where the mortality rate was 23% to 39.5% [8,36,38,40]. A mortality of just 14% was described where severe head injury accounted for only 32% of 22,924 patients [41]. Patients in our analysis with a pre-sedation GCS of 3 to 8 had a mortality of 38.5% compared to 44% mortality in a series of severe head injury only [27]. However, this included all hospital admissions whereas our analysis was restricted to those who were accepted for intensive therapy, and so would have included patients whose prognosis was deemed so dire and unmodifiable that they would not have been ICU candidates or who died prior to admission to an ICU.

It is notable that almost 30% of non-survivors in our analysis died after initial discharge from the ICU. This again may represent sound medical judgement in patients deemed to have a dire prognosis that would not improve significantly despite intensive care; however, only 8% of these patients had all active treatment withdrawn during their ICU stay, and only 19% were specified as a discharge for palliative care. Alternatively, it may again represent a lack of ICU provision to patients with great need for it. An estimated figure of 50% of non-survivors after surgery in the UK are never admitted to an ICU [31]. Clearly all of our patients were within an ICU at some point of their admission but it is possible that the length of time they spent there may not have been optimal.

Surgical status showed that approximately 20% of admissions required emergency surgery prior to their arrival in the ICU. However, the nature of the database does not inform us of the type of surgery performed and, although some may have undergone craniotomy, they may alternatively have undergone surgery for extracranial injuries, for example, laparotomy.

Our analysis also compared the performance of five risk prediction models in the prediction of head injury mortality in this population. Risk prediction models can be used to prognosticate but also to allow large-scale audit of outcomes in different centres or at different times. Observational studies of provision and outcomes in critical care often rely on risk prediction models to reduce bias. For these reasons, the models must be robust with as accurate calibration as possible to the particular population. Established models can display a loss of fit when evaluated in different critical care populations [42]. Even more so, this is a potential problem when they are evaluated in a single condition, such as head injury, for which they have not been specifically developed. We compared the models using a spectrum of measures of calibration and discrimination. This followed an approach developed under the guidance of an expert statistical steering committee for a large multicentre comparison of the risk prediction models in all ICU admissions [42]. The use of quantitative measures of model fit (Cox's calibration regression, Brier's score) rather than tests of perfect calibration (Hosmer-Lemeshow) alone allows more reliable comparison of the degree of miscalibration among the models. Although none of the risk prediction models evaluated in this analysis discriminated perfectly between survivors and non-survivors amongst 5,393 head injury patients in intensive care, SAPS II, MPM II and the ICNARC model discriminated better than APACHE II and III and had superior calibration. The performance of the risk prediction models surpassed that of raw GCS alone. This is a reflection of the importance of multiple factors in the prediction of outcome after head injury. Extracranial factors indirectly reflect the scale of secondary brain injury. Not only does the outcome from traumatic brain injury depend on adequate oxygenation and perfusion to facilitate restoration of normal neural architecture and physiology, insufficiency of these causes further neuronal insult. Therefore, incorporating the factors relating to systemic injury and cardiorespiratory function, for example, allows more accurate prediction of outcome.

The ICNARC model performed the best of all with respect to both discrimination and calibration. There may be several reasons for this. Firstly, this comparative analysis was based on an original dataset of 374,594 admissions from the CMP and the ICNARC model was derived using 231,930 of those admissions from the same database. Therefore, this would not be a fair representation of the ICNARC model's performance in head injury in intensive care. The comparative analysis was repeated using the remaining 142,664 admissions (2,563 head injuries), where none had been used to develop the ICNARC model, and it was again demonstrated to perform the best. A criticism that could still be raised is that these patients still have a similar case mix to those used to develop the ICNARC model as they came from the same UK ICUs. It is surprising, therefore, that the APACHE II model we used, which had been recalibrated for UK ICUs, did not perform better. In contrast, SAPS II and MPM II were developed using data from 137 ICUs in 12 countries throughout Europe and North America, the UK being only one of them, but still performed better than the UK-calibrated APACHE model. Thus, case mix can only partially explain the differences in model performance.

The second reason for the superior performance of the ICNARC model, followed by SAPS II and MPM II, may be their choice and weighting of variables relevant to neurological outcome. All of the models incorporate a mixture of the basic physiological factors that cause secondary brain injury, such as systolic blood pressure, hypoxia and temperature. However, they treat the neurological status of the patient differently and in varying depth. APACHE III uses a grid combining variations of eye opening, verbal and motor responses to give an overall score. The categories of each component are a compressed form of those in the Glasgow Coma Scale. SAPS II, on the other hand, uses the full GCS, which has been repeatedly shown to independently predict head injury mortality [6,8,26,43,44]. Although MPM II uses a cruder assessment of conscious level, that is, 'coma or deep stupor', it also incorporates the presence of intracranial mass effect. Presence of a mass lesion has also been demonstrated to be an independent predictor of head injury mortality [8,40,45,46]. In the case of sedated patients, APACHE II and III assume their GCS to be 15, which in the context of head injury requiring intensive care is clearly an often-false assumption and will underestimate disease severity. In contrast, SAPS II uses pre-sedation GCS as a direct replacement for the GCS in the first 24 hours, and the ICNARC model uses weightings for sedated and paralysed/sedated patients. The combination is much more likely to give a truer neurological assessment and make the ICNARC model a more appropriate tool in predicting head injury mortality.

## Conclusion

This study demonstrates that head injury patients requiring intensive care in the UK have a 77% chance of surviving to leave the ICU and a 66.5% chance of surviving to leave hospital. Non-survivors had a much briefer length of stay than survivors. When predicting mortality in this population using risk prediction models that have been successfully evaluated in the ICU, APACHE II and III were found to have poorer calibration and discrimination than the ICNARC model, SAPS II and MPM II. The ICNARC model performed the best of the five models evaluated, although all models had significant departures from perfect calibration. A comparison between the raw GCS and more detailed ICU predictive models demonstrated the better performance of the models and thus reflects the contribution of extracranial physiological factors to outcome after head injury. While the impact of individual physiological variables on outcome has been recognised by previous studies, we need to examine the relative contribution to outcome in our patient population.

## Key messages

• Traumatic brain injury requiring intensive care has an ICU mortality of 23.5%

• Length of stay is much shorter in-ICU and in-hospital for non-survivors

• The ICNARC model, SAPS II and MPM II have superior calibration and discrimination compared to APACHE II and III in traumatic brain injury, although none of these models have perfect calibration

• Lowest GCS from the first 24 hours in ICU had good discrimination when measured, but could not be objectively assessed for 57% of admissions

## Abbreviations

APACHE = Acute Physiology And Chronic Health Evaluation; AUC = area under the [ROC] curve; CMP = Case Mix Programme; GCS = Glasgow Coma Score; ICNARC = Intensive Care National Audit and Research Centre; ICU = intensive care unit; LOS = length of stay; MPM = Mortality Probability Models; ROC = receiver operating characteristic; SAPS = Simplified Acute Physiology Score.

## Competing interests

The authors declare that they have no competing interests.

## Authors' contributions

JH performed the literature review. CW performed the analyses. JH, CW and DH drafted the manuscript. All authors contributed to the design and interpretation of the study and critical revision of the manuscript, and have read and approved the final manuscript.

## References

[B1] Thurman DJ Alverson C Dunn KA Guerrero J Sniezek JE Traumatic brain injury in the United States: a public health perspective J Head Trauma Rehabil 1999 14 602 615 10671706 10.1097/00001199-199912000-00009

[B2] HESonline: Hospital Episode Statistics 2000–2001 http://www.hesonline.nhs.uk/

[B3] Watkins LD Head injuries: general principles and management Surgery 2000 18 219 224

[B4] Kay A Teasdale GM Head injury in the United Kingdom World J Surg 2001 25 1210 1220 10.1007/s00268-001-0084-6 11571960

[B5] Luerssen TG Klauber MR Marshall LF Outcome from head injury related to patient's age. A longitudinal prospective study of adult and pediatric head injury J Neurosurg 1988 68 409 416 3343613 10.3171/jns.1988.68.3.0409

[B6] Teasdale G Parker L Murray G Knill-Jones R Jennett B Predicting outcome of individual patients in the first week after severe head injury Acta Neurochir Suppl (Wien) 1979 28 161 164 158291 10.1007/978-3-7091-4088-8_39

[B7] Klauber MR Marshall LF Luerssen TG Frankowski R Tabaddor K Eisenberg HM Determinants of head injury mortality: importance of the low risk patient Neurosurgery 1989 24 31 36 10.1097/00006123-198901000-00005 2927596

[B8] Bahloul M Chelly H ben Hmida M ben Hmida C Ksibi H Kallel H Chaari A Kassis M Rekik N Bouaziz M Prognosis of traumatic head injury in South Tunisia: a multivariate analysis of 437 cases J Trauma 2004 57 255 261 15345970 10.1097/01.ta.0000083004.35231.1e

[B9] Chesnut RM Marshall LF Klauber MR Blunt BA Baldwin N Eisenberg HM Jane JA Marmarou A Foulkes MA The role of secondary brain injury in determining outcome from severe head injury J Trauma 1993 34 216 222 8459458 10.1097/00005373-199302000-00006

[B10] Alvarez M Nava JM Rue M Quintana S Mortality prediction in head trauma patient: performance of Glasgow Coma Score and general severity systems Crit Care Med 1998 26 142 148 10.1097/00003246-199801000-00030 9428557

[B11] Cho DY Wang YC Comparison of the APACHE III, APACHE II and Glasgow Coma Scale in acute head injury for prediction of mortality and functional outcome Intensive Care Med 1997 23 77 84 10.1007/s001340050294 9037644

[B12] Harrison DA Brady AR Rowan K Case mix, outcome and length of stay for admissions to adult, general critical care units in England, Wales and Northern Ireland: the Intensive Care National Audit & Research Centre Case Mix Programme Database Crit Care 2004 8 R99 R111 15025784 10.1186/cc2834 PMC420043

[B13] Young JD Goldfrad C Rowan K Development and testing of a hierarchical method to code the reason for admission to intensive care units: the ICNARC Coding Method Br J Anaesth 2001 87 543 548 10.1093/bja/87.4.543 11878722

[B14] Knaus WA Draper EA Wagner DP Zimmerman JE APACHE II: a severity of disease classification system Crit Care Med 1985 13 818 829 10.1097/00003246-198510000-00009 3928249

[B15] Knaus WA Wagner DP Draper EA Zimmerman JE Bergner M Bastos PG Sirio CA Murphy DJ Lotring T Damiano A Harrell FE Jr The APACHE III prognostic system. Risk prediction of hospital mortality for critically ill hospitalised adults Chest 1991 100 1619 1636 1959406 10.1378/chest.100.6.1619

[B16] Le Gall JR Lemeshow S Saulnier F A new Simplified Acute Physiology Score (SAPS II) based on a European/North American multicentre study JAMA 1993 270 2957 2963 10.1001/jama.270.24.2957 8254858

[B17] Lemeshow S Teres D Klar J Avrunin JS Gehlbach SH Rapoport J Mortality Probability Models (MPM II) based on an international cohort of intensive care unit patients JAMA 1993 270 2478 2486 10.1001/jama.270.20.2478 8230626

[B18] Harrison DA Parry GJ Carpenter JR Short A Rowan K A new risk prediction model for critical care: the Intensive Care National Audit & Research Centre (ICNARC) model Intensive Care Med 2006 32 Suppl 1 S204 10.1097/01.CCM.0000259468.24532.44 17334248

[B19] Rowan KM Outcome comparisons of intensive care units in Great Britain and Ireland using the APACHE II method DPhil thesis 1992 Department of Public Health and Primary Care, University of Oxford

[B20] Hanley JA McNeil BJ The meaning and use of the area under a receiver operating characteristic (ROC) curve Radiology 1982 143 29 36 7063747 10.1148/radiology.143.1.7063747

[B21] Hosmer DW Jr Lemeshow S Goodness-of-fit tests for the multiple logistic regression model Commun Stat 1980 A9 1043 1069 10.1002/(sici)1097-0258(19970515)16:9<965::aid-sim509>3.0.co;2-o 9160492

[B22] Cox DR Two further applications for a method of binary regression Biometrika 1958 45 562 565 10.2307/2333203

[B23] Brier GW Verification of forecasts expressed in terms of probability Monthly Weather Review 1950 75 1 3

[B24] MacLeod JB Lynn M McKenney MG Cohn SM Murtha M Early coagulopathy predicts mortality in trauma J Trauma 2003 55 39 44 12855879 10.1097/01.TA.0000075338.21177.EF

[B25] Lane PL Skoretz TG Doig G Girotti MJ Intracranial pressure monitoring and outcomes after trauma brain injury Can J Surg 2000 43 442 448 11129833 PMC3695200

[B26] Demetriades D Kuncir E Murray J Velmahos GC Rhee P Chan L Mortality prediction of head Abbreviated Injury Score and Glasgow Coma Scale: analysis of 7,764 head injuries J Am Coll Surg 2004 199 216 222 10.1016/j.jamcollsurg.2004.02.030 15275876

[B27] Patel HC Bouamra O Woodford M King AT Yates DW Lecky FE Trends in head injury outcome from 1989 to 2003 and the effect of neurosurgical care: an observational study Lancet 2005 366 1538 1544 10.1016/S0140-6736(05)67626-X 16257340

[B28] O'Jile JR Ryan LM Parks-Levy J Betz B Gouvier WD Sensation seeking and risk behaviours in young adults with and without a history of head injury Appl Neuropsychol 2004 11 107 112 10.1207/s15324826an1102_7 15477182

[B29] Jennett B Teasdale G Braakman R Minderhoud J Knill-Jones R Predicting outcome in individual patients after severe head injury Lancet 1976 15 1031 1034 10.1016/S0140-6736(76)92215-7 57446

[B30] Jennett B Teasdale G Braakman R Minderhoud J Heiden J Kurze T Prognosis of patients with severe head injury Neurosurgery 1979 4 283 289 10.1097/00006123-197904000-00001 450225

[B31] Grocott MPW Ball JAS Consensus meeting: management of the high risk surgical patient Clin Intensive Care 2000 11 263 281

[B32] Bennett-Guerrero E Hyam JA Shaefi S Pryterch DR Sutton GL Weaver PC Mythen MG Grocott MP Parides MK Comparison of P-POSSUM risk-adjusted mortality rates after surgery between patients in the USA and the UK Br J Surg 2003 90 1593 1598 10.1002/bjs.4347 14648741

[B33] Department of Heath NHS Reference Costs 2005 2006 London: Department of Heath

[B34] Metcalfe MA Sloggett A McPherson K Mortality among appropriately referred patients refused admission to intensive care units Lancet 1997 350 7 11 10.1016/S0140-6736(96)10018-0 9217712

[B35] Bion J Rationing intensive care BMJ 1995 310 682 683 7711527 10.1136/bmj.310.6981.682 PMC2549089

[B36] Clayton TJ Nelson RJ Manara AR Reduction in mortality from severe head injury following introduction of a protocol for intensive care management Br J Anaesth 2004 93 761 767 10.1093/bja/aeh249 15347602

[B37] Gupta D Keogh B Chung KF Ayres JG Harrison DA Goldfrad C Brady AR Rowan K Characteristics and outcome for admissions to adult, general critical care units with acute severe asthma: a secondary analysis of the ICNARC case mix programme database Crit Care 2004 8 R112 R121 15025785 10.1186/cc2835 PMC420044

[B38] Bulger EM Nathens AB Rivara FP Moore M MacKenzie EJ Jurkovich GJ Management of severe head injury: institutional variations in care and effect on outcome Crit Care Med 2002 30 1870 1876 10.1097/00003246-200208000-00033 12163808

[B39] Wildman MJ Harrison DA Brady AR Rowan K Case mix and outcomes for admissions to UK adult, general critical care units with chronic obstructive pulmonary disease: a secondary analysis of the ICNARC Case Mix Programme Database Crit Care 2005 9 suppl 3 S38 S48 10.1186/cc3719

[B40] Levati A Farina ML Vecchi G Rossanda M Marrubini MB Prognosis of severe head injuries J Neurosurg 1982 57 779 783 7143060 10.3171/jns.1982.57.6.0779

[B41] Udekwu P Kromhout-Schiro S Vaslef S Baker C Oller D Glasgow Coma Scale score, mortality, and functional outcome in head-injured patients J Trauma 2004 56 1084 1089 15179250 10.1097/01.ta.0000124283.02605.a5

[B42] Harrison DA Brady AR Parry GH Carpenter JR Rowan K Recalibration of risk prediction models in a large multicenter cohort of admissions to adult, general critical care units in the United Kingdom Crit Care Med 2006 34 1378 1388 10.1097/01.CCM.0000216702.94014.75 16557153

[B43] Lang EW Pits LH Damron SL Rutledge R Outcome after severe head injury: an analysis of prediction based upon comparison of neural network versus logistic regression analysis Neurol Res 1997 19 274 280 9192380 10.1080/01616412.1997.11740813

[B44] Edna TH Risk factors in traumatic head injury Acta Neurochir (Wien) 1983 69 15 21 10.1007/BF02055848 6624551

[B45] Narayan RK Greenberg RP Miller JD Enas GG Choi SC Kishore PR Selhorst JB Lutz HA 3rd Becker DP Improved confidence of outcome prediction in severe head injury. A comparative analysis of the clinical examination, multimodality evoked potentials, CT scanning, and intracranial pressure J Neurosurg 1981 54 751 762 7241184 10.3171/jns.1981.54.6.0751

[B46] Stablein DM Miller JD Choi SC Becker DP Statistical methods for determining prognosis in severe head injury Neurosurgery 1980 6 243 248 10.1097/00006123-198003000-00003 6770283

[B47] ICNARC: Participating Units http://www.icnarc.org/audit/cmp/participating-units/

